# Coprophagia in an 8-Year-Old Hospitalized Patient: A Case Report and Review of the Literature

**DOI:** 10.1155/2017/6565096

**Published:** 2017-11-27

**Authors:** Aleksandra Bacewicz, Katherine Martin

**Affiliations:** ^1^University of South Florida College of Medicine, Tampa, FL, USA; ^2^Lehigh Valley Health Network, Allentown, PA, USA

## Abstract

Consult-liaison psychiatrists often encounter difficult clinical scenarios. We present a pediatric case of presumptive coprophagia. After a negative medical work-up, the pediatrics team asked psychiatry to assist them in managing this relatively rare disorder in the hospital setting. Little is known about the etiology and treatment of coprophagia in the pediatric population. Using the case as a catalyst, we discuss what is known about this disorder as well as treatment strategies in the hospital setting.

## 1. Introduction

Coprophagia is a relatively rare disorder associated with neurological and psychiatric disorders. We present the case of an 8-year-old male patient admitted to the pediatric service who presented with feculent emesis of well-formed stool. He had a normal GI work-up, which revealed no fistulas. While observed, he had no further episodes. When left alone for a brief period of time, his symptoms resumed. Fingermarks were found in the toilet hat used to collect his stool and a presumptive diagnosis of coprophagia was made. We review the underlying disorders associated with coprophagia as well as its behavioral management in a general hospital setting.

## 2. Case Report

An 8-year-old male presented for one week of feculent emesis. In the week prior to his presentation at our institution, he had had multiple visits to his pediatrician and outside emergency departments for similar complaints. He had been held overnight at an outside hospital. While there, he had a normal abdominal X-ray and cat scan. He was discharged with a diagnosis of constipation and was started on MiraLax and Senna.

When he presented to our institution, his mother reported that he had continued to have feculent emesis of well-formed stool about 5-6 times per day. He was admitted to the pediatric service and did not have any further episodes of emesis. He had a normal physical exam, was very well appearing, and did not seem upset by his symptoms. He furthermore had a normal abdominal X-ray and a normal upper GI series with fluoroscopy. His bloodwork was also nonrevealing and he had a normal CBC and CMP. His stool testing was negative for occult blood. He was seen by the pediatric gastroenterology service that commented that it would not be possible to vomit well-formed stool in the absence of a large colon to gastric fistula, but then he would also be expected to have diarrhea and weight loss, which he did not.

On hospital day number three, he was left alone briefly for the first time when his mother left the room to get lunch. During this time period, he reported his first episode of fecal emesis. When staff went into the room to investigate, it was noted that there were smear marks on the toilet hat and a strong suspicion that he had eaten the fecal matter. He was placed on 1 : 1 observation and a psychiatry consult was ordered.

The psychiatric consult-liaison team interviewed the patient and his mother separately. He was born full term and had met his milestones appropriately. He had no significant medical history and his surgical history included an adenoidectomy. His prior psychiatric history consisted of one year of outpatient counseling after the death of his father four years earlier from a pulmonary embolus. However he had been stepped down to case management because he had been doing well. He had never been diagnosed with an intellectual disability, but he did have an individualized education program that specified he required extra help with reading. His mother reported he had a history of an oral fixation and frequently put items in his mouth. She also reported he was immature for his age, tended to have younger friends, and was repeating second grade for this reason. He denied being bullied at school. He lived with his mother and older brother and denied any history of abuse in the past or currently. He did have a history of his house being flooded and having to move two years prior. He denied any illicit substance use. His family psychiatric history consisted of his mother reporting a history of borderline personality disorder and PTSD related to a prior sexual assault while serving in the military. Both the patient and his mother denied any acute stressors connected to his symptoms. His mood was “good” and his affect was bright. He did not appear bothered by his symptoms in the least. He was asked directly if he had consumed his feces on several instances and he consistently denied it, stating that that would be “gross.”

He remained on a 1 : 1 for the remainder of his admission and had no further episodes. It was recommended that he intensify his outpatient psychiatric treatment where he had been seen in the past and currently had a case manager. His mother had a very difficult time accepting the presumptive diagnosis of coprophagia but was agreeable to the recommendation for increased outpatient psychiatric services. He was subsequently discharged, but on the way home from the hospital, he began having further episodes. He was brought back to the emergency department and admitted overnight but had no more feculent emesis. He subsequently was discharged with the same recommendations.

## 3. Discussion

Some researchers have classified the act of eating one's own feces, coprophagia, as an unusual form of pica [1, 2]. Pica is defined as “the persistent ingestion of nonnutritive substances for more than one month at an age when this behavior is deemed inappropriate” [3]. However, there is no agreement among researchers that coprophagia is a variant of pica. Some researchers argue that coprophagia is not and that patients with coprophagia do not manifest other hyperphagic behaviors that could be the case with pica [4]. As with pica, the etiology of coprophagia is neither well-understood nor well-researched within the adult or pediatric population. There is a particular paucity of knowledge available about the presence of coprophagia in pediatric patients.

Coprophagia has been studied in animals. For dogs, it seems to be related to boredom, as well as avoiding punishment for defecating inside the house. Coprophagia can be considered normal for puppies, as they shed this behavior when they enter adolescence [5]. Certain rabbits and rodents also manifest this behavior, likely for a primarily nutritive purpose [6]. Studies have shown that coprophagia is possible to induce through experimental thiamine deficiency in dogs and by total lesions of the amygdala in primates. However, experimental thiamine deficiency trials have only been done on animals and coprophagia has been found to be present in human patients with no thiamine deficiencies [7].

In humans, the causes of coprophagia are considered to be broad and uncertain. The Mayo Clinic clinical database was queried for all patients evaluated between 1995 and 2015 in which coprophagia was mentioned. Seventeen patients had coprophagia and 12 were adults that were included in the study. The median age at onset was 55 years (range 20–88 years), and half were females. Additional behaviors were common including scatolia (fecal smearing), hypersexuality, aggression, and pica (eating objects of any kind). In this study, coprophagia was associated with neurodegenerative dementia in six patients, developmental delay in two and one each with seizures, steroid psychosis, frontal lobe tumor, and schizoaffective disorder [6]. Case studies have identified additional disorders associated with coprophagia, including autism spectrum disorders, schizophrenia, epilepsy, fetishism, OCD, depression, and organic brain disease. Coprophagia in children with developmental delay appears to be related to a lack of proper toileting skills [2].

A posited etiology of coprophagia is structural brain change, specifically damage to the bilateral amygdala. Bilateral amygdalar damage in both humans and animals causes Kluver-Bucy syndrome, which encompasses behaviors of hyperorality and hyperphagia. However, in the 12 adult patients exhibiting this behavior in the Mayo Clinic clinical database study, there was no consistent brain lesion found on imaging. The six patients with dementia demonstrated signs of medial temporal lobe atrophy and frontal lobe atrophy as could be expected with their condition, while patients with other organic brain diagnoses were only found to have lesions correlating to their primary problem [6]. Additionally, one prior case report, among others, describes the development of coprophagia in a 46-year-old male with frontotemporal multiform glioblastoma and no evidence of amygdalar damage.

Although brain imaging was not done, there was nothing in the history and physical of our patient that would suggest brain damage [7].

Our patient had met his milestones growing up and had not been diagnosed as developmentally delayed. However, his mother described him as needing extra assistance in school with reading and as immature for his age, forcing him to repeat the second grade and suggesting that he did struggle academically. Additionally, his mother described him as having an oral fixation. In one of the few prior pediatric case studies, a 6-year-old child with autism was found to develop coprophagia, as well as associated behaviors of fecal smearing and self-injurious behavior [1].

However, our patient was not known to have an autism spectrum disorder.

The patient's mother in this case report had a history of PTSD due to prior sexual abuse, as well as borderline personality disorder. The child denied any past or current abuse and his mother did not note any acute stressors at the time of onset of this behavior. He had received outpatient counseling for one year, as his father had died four years prior to this presentation. Additionally, the patient did have a history of his home being flooded and having to move two years before.

There have been isolated case studies of coprophagia manifesting as a response to guilt or anxiety. In one such case, a 20-year-old man, also a fourth-year university student with an A− average, presented with coprophagia. He had been forced to eat his feces as punishment when he was a child and later continued to do so after separate instances of guilt over his mother's death, a motor vehicle accident, and an unwanted pregnancy and abortion in his girlfriend [8]. He was in noticeable distress at the time he was evaluated and described “unending” anxiety if he did not engage in this behavior. Our patient, interestingly, did not show any distress on mental exam and appeared unaffected by his behavior.

Some of the studied cases of coprophagia have been in individuals with psychiatric histories that include an affective disorder, such as depression. For example, an 83-year-old female with Alzheimer's dementia and major depressive disorder was found to have coprophagia [9]. She had a history of one suicide attempt and had been seeing a geriatric psychiatrist three weeks prior to the diagnosis with coprophagia.

Another case study linked coprophagia to a paraphilia in addition to depression. In this example, a 47-year-old male, who was nonpsychotic and had normal intelligence, exhibited behaviors of scatolia and coprophagia, in association with masturbation. He was also suffering with depression and alcohol use. Ultimately, he was found to have resolution of both his depression and coprophagia with alcohol abstinence, joining Alcoholics Anonymous, supportive psychotherapy, and a tricyclic antidepressant [10].

Our patient was ultimately recommended to increase his outpatient psychiatric services after the presumptive diagnosis of coprophagia was made. The mainstay of treatment for coprophagia is behavioral, but pharmacotherapy has been effective in some instances. In a case report of a 29-year-old male with high functioning autism spectrum disorder and no history of difficulties with toilet training or significant bowel disease, changing from risperidone 0.5 mg BID to aripiprazole 15 mg markedly reduced symptoms [11]. In a 46-year-old male with left frontotemporal multiform glioblastoma, carbamazepine 300 mg TID with serum level of 7.8 ug/mL was effective at ceasing the behavior [7].

In the patients included in the Mayo Clinic search, medications tried included lorazepam, citalopram, trazodone, mirtazapine, quetiapine, valproic acid, and haloperidol. Haloperidol, in oral doses from 1 to 3 mg daily, eliminated coprophagia in four of the patients. The other medications as monotherapy or in combination with others were not found to be effective [6].

Behavioral intervention has been attempted in patients with coprophagia and has been found to be successful in some. In a 2005 literature review of patients with coprophagia, the patients described presented with this behavior over a broad range of circumstances and diagnoses. Among individuals with intellectual disability and a diagnosis of coprophagia, patients benefited separately from an elemental diet (although it was not specified which nutrient was lacking) and aversion therapy. Aversion therapy included cleaning of the mouth, hand washing, and clearing the patient's surroundings of potential objects of ingestion. Reinforcement of toileting skills and regular toileting were also mentioned as potential treatments. In a patient with obsessive-compulsive disorder (OCD), exposure and response therapy was effective after about two months; the patient denied coprophagia at one-year follow-up [2].

## 4. Conclusions

The etiology of coprophagia remains unclear, but it has been associated with multiple disorders, including dementia, developmental delay, steroid psychosis, autism spectrum disorders, schizophrenia, epilepsy, fetishism, OCD, brain tumors, depression, and organic brain disease [6]. Therefore, when it is encountered in a clinical setting, a careful history should be elicited to assess for any of these underlying disorders. In the care of our patient, it was also necessary to rule out a gastric fistula given his history of feculent emesis.

In a general hospital setting, it is advised that patients be placed on a 1 : 1 while ruling out medical causes. This step was extremely helpful in confirming the diagnosis in our patient. Once the presumptive diagnosis of coprophagia is made, the diagnosis should be conveyed in a nonjudgmental way by the primary medical team, but a consult-liaison psychiatrist should be on hand to process the diagnosis with the patient and family and make treatment recommendations.

While the mainstay of treatment for coprophagia is behavioral therapy, depending on the underlying etiology, there may be certain instances when pharmacology may be appropriate. For example, it would be appropriate to use an antipsychotic for a patient with schizophrenia, an antiepileptic for a patient with epilepsy, and antidepressant for a patient with a depressive disorder. Given the relative paucity of pediatric clinical cases of coprophagia, recommendations would have to be made on a case-by-case basis, informed by the suspected underlying etiology of coprophagia.
